# A case of high-grade leiomyosarcoma of the bladder with delayed onset and very poor prognosis

**DOI:** 10.1186/1477-7819-8-16

**Published:** 2010-03-19

**Authors:** Enzo Ricciardi, Paolo Maniglio, Mauro Schimberni, Massimo Moscarini

**Affiliations:** 1 Department of Gynecology, Perinatology and Child Health. Sapienza University of Rome. Sant'Andrea Hospital, Via di Grottarossa 1035-1039, 00195, Rome, Italy

## Abstract

Mesenchymal tumors represent a small number of bladder cancer cases. Leiomyosarcoma is the most common histology with over 100 cases reported in the whole literature. This tumor is been historically considered as highly aggressive and showing a poor prognosis. Despite very low survival rates showed in older reports, some authors indicate that some patients could have a better outcome. We report a review of the literature and a case of high-grade LMS of the bladder in a 68 years old woman. Diagnosis was delayed and disease was locally advanced. Symptoms and imaging of our case first oriented to a gynecologic condition with an adnexal or uterine origin of the mass, and, a genitourinary origin could be unveiled only intra-operatively.

## Background

Non-epithelial tumors of the urinary bladder account for less than 5% of the overall bladder malignancies, with leiomyosarcoma being the 0.1% of bladder cancer [1]. There are over 100 cases reported in the whole medical literature as leiomyosarcomas, and a total of 192 cases considering all bladder sarcoma cases [2]. There is a lack of consensus about a standard treatment, and little is known about natural history and prognosis of the tumor, due to a very low incidence.

Cases presented in literature had mostly gross urinary symptoms, more often with an earlier onset. This may, eventually, lead to earlier diagnosis, allowing a safer therapeutic approach. The case we report shows an unusual presentation, differing from the symptoms reported in literature, resembling mostly a pelvic mass. The patient underwent, in the early diagnostic steps, a gynecologic evaluation that addressed to a final multidisciplinary surgical approach to the disease. The high-grade and stage of the tumor, in association to the fatal outcome of the case, are expression of the aggressive behavior of the tumor, showing mostly a very poor prognosis.

## Case presentation

We report a case of a 68 years old woman presenting to the emergency department with macrohematuria and dysuria. She experienced fever several times, starting two months before medical examination. Fever was treated with NSAID. A history of recent pelvic pain was also reported. Her clinical status was good and no other symptoms or concurrent illnesses were present at the time of hospitalization.

She was admitted to Gynecologic service for further evaluation.

The patient was virgin and in post-menopausal status. Clinical history was unremarkable; negative either for previous medical or surgical procedures. Family history was positive for cancer with one male sibling deceased from a pancreatic malignancy; the remainder two siblings had no positive history for malignancies as well as parents and closest relatives. Social history was negative for use or addiction to drugs, alcohol and exposure to potential risk factors.

A digital rectal examination had to be performed in place of the gynecologic bimanual examination. A gross, firm mass located in the pelvis was found at physical examination.

Pelvic US, abdominal CT scans, descending pielography, cystoscopy and chest RX were performed to both assess local disease and evaluate the presence of local and distant metastases.

Computed tomography imaging confirmed the presence of a complex mass in the pelvis (13 × 14 cm) showing irregular contours and small areas of calcifications. Uterus and adnexa could not be clearly recognized, showing as being part of the mass. Iliac vessels and bladder were displaced. Imaging technique reports stated that no vessels or bladder invasion was present. A hypoplasia of the left kidney was also reported. No other metastasis was reported in the abdomino-pelvic cavity.

Pielography showed a delay in the opacification of the renal pelvis associated to a dilation of the right ureter that was compressed and displaced in its pelvic course till entering the bladder. No opacification of the contra lateral kidney and excretory ways could be obtained.

Cystoscopy showed, eventually, a bleeding lesion onto the bladder wall that raised suspicion of neoplastic invasion. The lesion appeared more likely as a loss of continuity of the urothelial mucosa; an ulcerating mass was visualized and eventually described on final report.

Chest RX and bone scan were negative for distant metastases.

A multidisciplinary board, including a urologist, a radiologist, a radiation oncologist, an oncologist and a gynecologist, evaluated the collected data. It was eventually decided to surgically extirpate the tumor. A transurethral biopsy of the vesical wall lesion was not considered since major suspicion was addressing to uterine or adnexal neoplasms and the high stage was reasonably an indication to undergo definitive surgery.

Surgery was eventually performed after pre-operative routine and evaluation.

A longitudinal abdominal incision was preferred to access peritoneal cavity. A bulky mass was found in the pelvis. Uterus and adnexa appeared macroscopically free from neoplastic invasion. The mass showed a cleavage with recto-sigmoid tract, iliac vessels and pubic bone, whether uterus, adnexa, right ureter and left obturator nerve could not be separated from the tumor. A histological intra-operative examination revealed a malignant, spindled cell neoplasm. Surgery had to include a wide mass excision, in order to obtain free margins and control of local disease. Final treatment choice was a pelvic exenteration. This decision was made since local disease was advanced and it was set to obtain specimen's margins disease-free as a primary target.

An en-bloc resection of the mass, together with uterus, adnexa, bladder, distal part of right ureter and left obturator nerve was performed (anterior pelvic exenterantion).

A cutaneous ureterostomy was placed in the site of ureteral dissection. The decision to perform this procedure in place of a standard urinary diversion, as a ileal conduit or Miami pouch, was made on the evidence of poor prognosis that appeared from surgical exploration and intra-operative histology that was addressing to a malignant stromal neoplasm. It seemed to be reasonable to lower intra- and post-operative morbidity whereas the potential overall survival of the patient appeared to be prospectively low.

**Figure 1:**
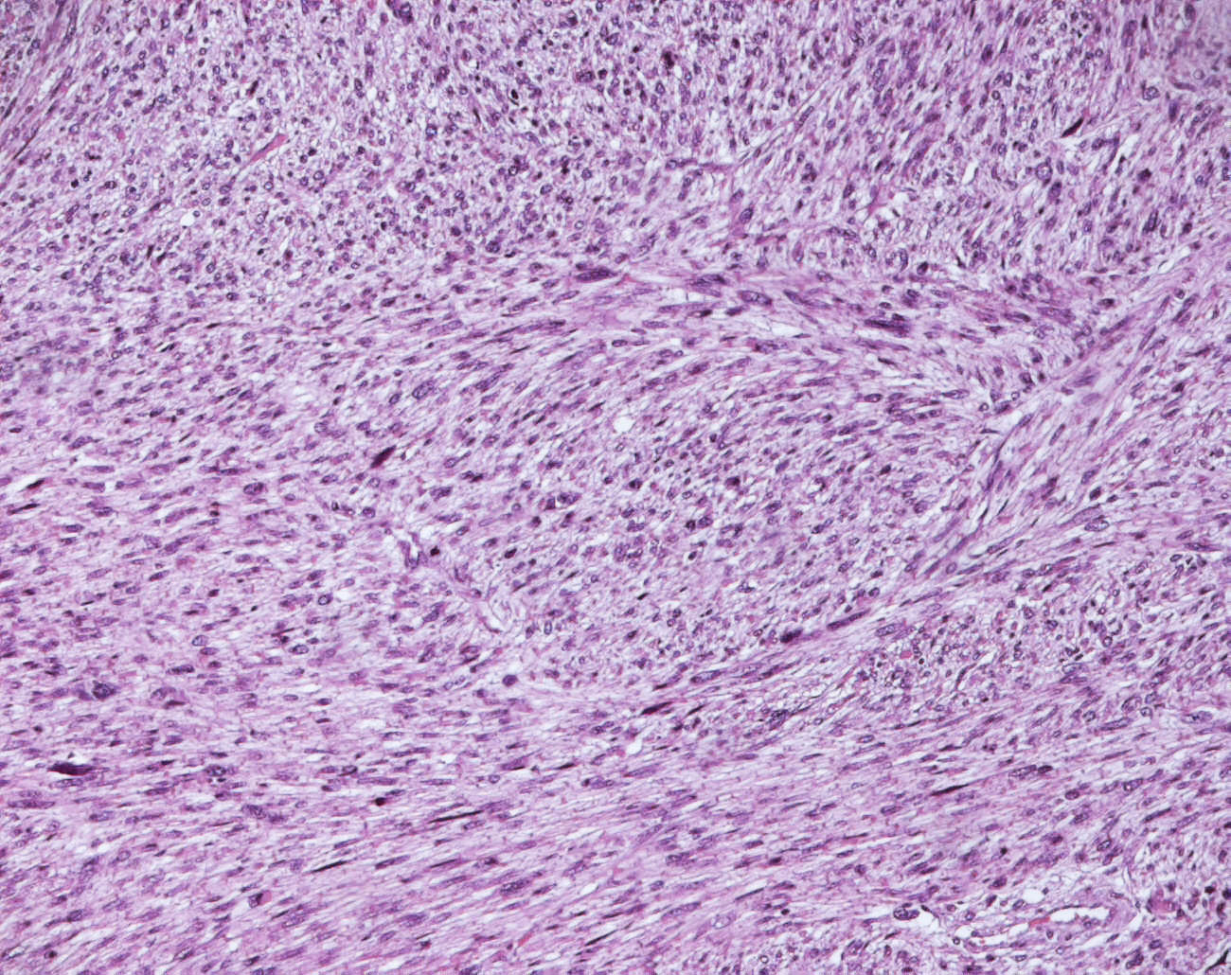
**Leiomyosarcoma of the bladder (Hematoxylin and eosin)**.

**Figure 2:**
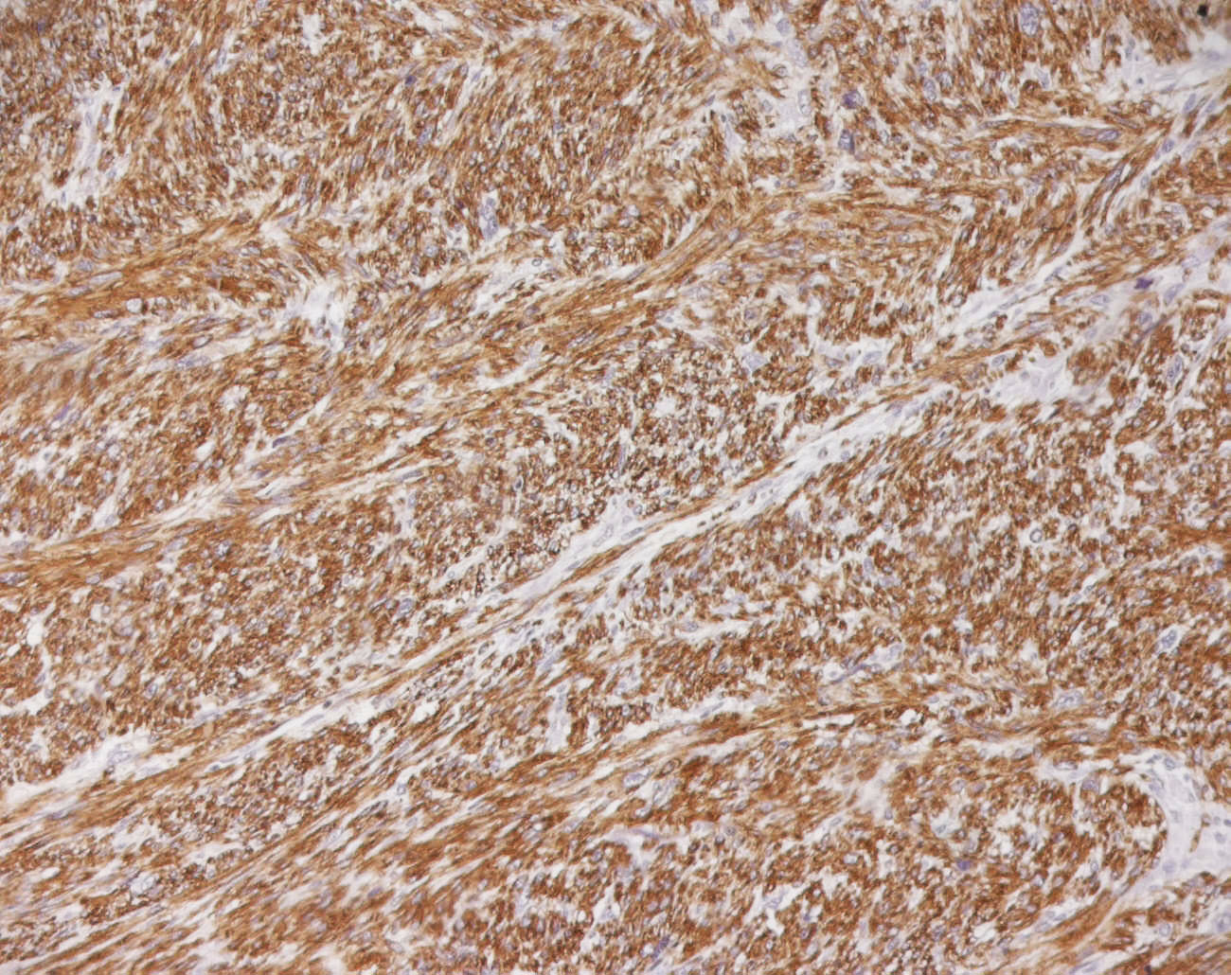
**Leiomyosarcoma of the bladder (Smooth muscle actin)**.

**Figure 3:**
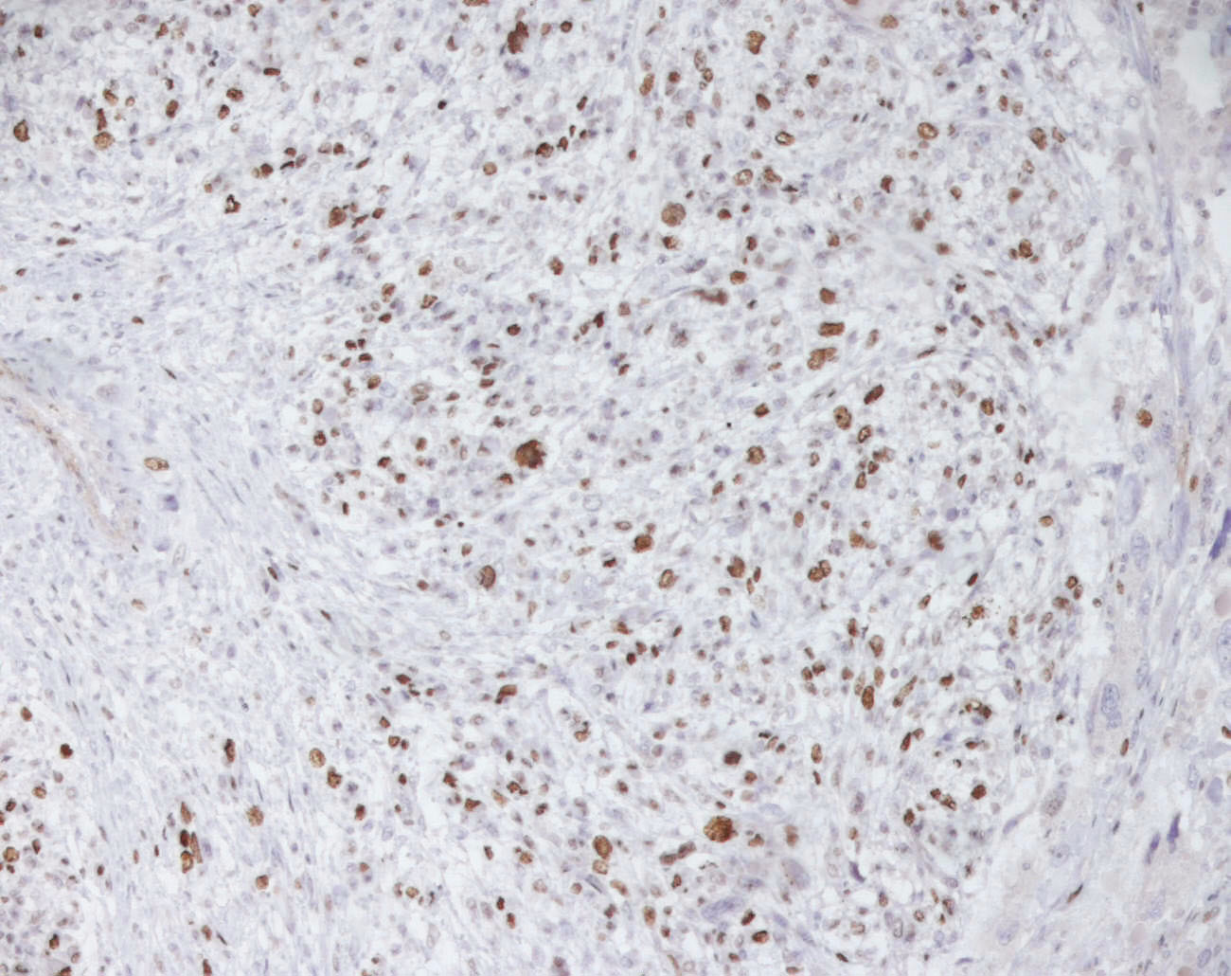
**Leiomyosarcoma of the bladder (Ki67)**.

A final histological diagnosis of high-grade leiomyosarcoma, (Fig. 1), (G3, FNCLCC 1986) pT2b pN0 pM0, AJCC (2002) Stage III, was made based on immunoreactivity to smooth muscle actin, score 2 (Fig. 2). The specimen resulted negative to c-kit and EGFR. Proliferation index was 90%, evaluated thru MIB-1 (Ki-67) (Fig. 3). Necrosis was inferior to 50% (score 2) and mitotic index higher than 20 mitosis per field/10 HPF (score 3). The tumor presented, macroscopically, as a bulky, brain-like, white-grayish mass. The tumor contained diverse necrotic and hemorrhagic areas, and invaded the vesical wall. The size of the mass was 14 × 11 × 6.5 cm. Histology confirmed that uterus and adnexa were free from neoplastic invasion. Surgical margins resulted negative at the final examination. LVI was not reported.

Post-operative course was free from complications and the patient could be discharged from the hospital fourteen days after surgery.

The patient underwent chemotherapy one month after surgery, using doxorubicin as a single agent in an adjuvant setting. She died after the first cycle from a distant recurrence to the left lung.

## Discussion

Sarcoma is the most frequent mesenchymal malignancy of the bladder with leiomyosarcoma as most common histology, according to two retrospective reviews of mesenchymal genitourinary tumors [3,4]. Considering the whole of the cases reported in literature (192 in total), 50% were leiomyosarcomas, 20% rhabdomyosarcomas and the remainder consisting of other histologies as carcinosarcomas, angiosarcomas and osteosarcomas [2]. No specific risk factors have been identified yet for this tumor. Reviewing the literature, retinoblastoma (RB) gene mutations were identified among possible causes in at least 9 cases, as well as use of cyclophosphamide [5-8]. Pelvic radiation therapy for other malignancies was also described in literature as a risk factor present in the clinical history of the patient. According to Rosser et al., the most common clinical presentation is gross hematuria (81%), dysuria (19%) and pollakiuria (28%). Our case presented mostly as a pelvic mass, showing urinary symptoms similar to those described by Rosser and coll [9] but very late in disease course. Diagnosis in our case was therefore delayed, due on the fact that the patient had no symptoms until tumor reached an advanced stage and became locally invasive. The patient, otherwise being well, did not consider undergoing a medical evaluation, until the onset of urinary symptoms.

Initial reports for this tumor showed a very aggressive disease. In a series by Mackenzie et al. published in 1968, only 11 patients were alive at 3 years from surgery [10]. Further series showed that the outcome shown in previous series, could be better that once believed. A series at MD Anderson considered 19 patients with a bladder sarcoma diagnosis. It showed a 5 years disease-specific survival of 59%. They also reported lymph-vascular invasion and node status as significant prognostic markers, claiming a 3.4 median survival for patients with no LVI in front of a 0.8 median survival when LVI was present. Pathologic node status showed a median survival of 7.6 when negative and 0.3 when positive [11]. Largest series, by Rosser and coll. (35 patients, with a diagnosis of LMS of the bladder) showed a 5 years disease-specific survival of 62%, with a 34% recurrence rate at a median follow-up of 38 months, with both local and distant disease sites affected [9].

However, the best prognostic factor seems to be the presence of free margins. In our case, we achieved disease-free margins; still, they did not determine a better outcome. In addition to margin status, local invasiveness and size, as well as tumor grade, seem to play an important role in determining the final outcome. In our case, as stated above, LVI was unavailable.

According to one of the greatest series available to date, overall local recurrence of bladder leiomyosarcomas is about 16%, with most recurrences occurring in the pelvis. Overall recurrence of distant metastases is about 53%, with the most common sites of metastases being the lungs, liver, bone, and brain [11].

Because of very low incidence, there is no universal consensus on the treatment of patients affected. Minimally invasive approaches, such as transurethral resection or laser fulguration, in addition to CTX or RT adjuvant settings, have been used for patients with small lesions. Long-term survival rates did not differ significantly to cases undergone a more radical surgery. A sole resection of the mass, even when free margins were achieved, as in case of partial cystectomy, should be considered a palliative treatment [12-14]. Main treatment consists in radical cystectomy (including removal of uterus, cervix and vaginal cuff in women). The procedure should include wide margins resection with 2-3 cm depth free from tumor invasion [15-17,19]. Neoadjuvant and adjuvant therapies were used in 21% and 16% of patients at MD Anderson, respectively, and both resulted in a doubling of disease-specific survival. However, this result was not statistically significant, reflecting the small numbers of patients in each group. Similarly, it is difficult to evaluate the impact of neoadjuvant and adjuvant chemotherapy on quality of life [11].

A multimodal treatment with CTX settings targeting mesenchymal cancers (as sarcoma chemotherapy protocols using doxorubicin, ifosfamide, cisplatinum and docetaxel) should be mandatory in the event of metastatic disease, [11]. With positive margins after surgery, adjuvant radiotherapy should be advocated for the patient [18]. Local recurrences should be treated by systemic CTX and/or external pelvic RT, salvage therapy showed to be ineffective, with a median survival of 20 months after surgery [11].

## Conclusions

Sarcomas should be considered as a possible histology at differential diagnosis, even if they are no frequent compared to other tumors. Unfortunately, obtaining a pre-operative histological diagnosis does not improve the prognosis. Rare tumors represent a great challenge for physicians. They require experienced teams and well-equipped centers for cancer cure. Bladder sarcomas, as other genito-urinary sarcomas, require close cooperation between urologist and gynecologic oncologist, as well as medical and radiation oncologist. A pathologist expert in mesenchymal tumors is mandatory.

Nowadays, no statistically relevant evidences on therapeutic behavior can be found yet in the literature. Therefore, treatment should be tailored case-by-case, preferring a multimodal and/or multidisciplinary approach to the disease. A decision-making team made by physicians experienced in managing soft tissues sarcomas should be also strictly required.

## Competing interests

The authors report no conflicts of interest. The authors alone are responsible for the content and writing of the paper.

## Authors' contributions

ER conceived of the study, and participated in its design and coordination and drafted the manuscript. PM collected clinical data. MS participated in collecting data and read and corrected the manuscript. MM participated in design of the study and revisions, gave intellectual input and corrected the manuscript. All authors read and approved the final manuscript.

## Consent

Written informed consent was obtained from the patient for publication of this case report and any accompanying images. A copy of the written consent is available for review by the Editor-in-Chief of this journal.
